# CD34‐TdT‐B‐ALL masquerading as Burkitt lymphoma

**DOI:** 10.1002/jha2.884

**Published:** 2024-04-21

**Authors:** Manu Juneja, Andrew Wei, Kylie Mason, Tamia Nguyen, John F Seymour, Surender Juneja

**Affiliations:** ^1^ Department of Clinical Haematology Peter MacCallum Cancer Centre and Royal Melbourne Hospital Melbourne Australia; ^2^ Division of Blood Cells and Blood Cancer Walter and Eliza Hall Institute Melbourne Australia; ^3^ Faculty of Medicine, Dentistry and Health Sciences University of Melbourne Melbourne Australia

**Keywords:** ALL, Burkitt lymphoma, WHO 22

1

A 23‐year‐old female presented with widespread petechiae and oral mucosal bleeding. There was no palpable lymphadenopathy or hepatosplenomegaly. Complete blood count included haemoglobin 77 g/L (normal 115–155), white cell count 20.4 × 10^9^/L (4.0–12.0), “blasts” 10.6 (52%), neutrophils 3.3 × 10^9^/L (2.0–8.0), platelets 11 × 10^9^/L (150–400). Peripheral blood morphology demonstrated monomorphic immature mononuclear cells with deeply basophilic cytoplasm and numerous small cytoplasmic vacuoles (Figure 1, left image; 1000x magnification) and markedly elevated serum lactate dehydrogenase (14358 IU; 120–250), suggestive of Burkitt leukaemia/lymphoma. Blood was analysed using standard methods as outlined by the ICSH (International Committee for Standardization in Haematology). Immunophenotyping demonstrated CD10+, CD19+ CD20+, CD22+,CD24+, CD34‐, CD38+, CD45+ (dim) and CD58+ with cytoplasmic lambda light chain restriction (without surface light chain expression) consistent with a B‐cell lymphoma or pre‐B ALL. Bone marrow was markedly hypercellular with 83% neoplastic cells with identical morphology (Figure 1, middle image; 1000x magnification). Immunohistochemistry was negative for TdT and CD34. Chromosomal studies identified an unbalanced t(5;8)(q13;p21) resulting in the loss of part of the long arm of chromosome 5, and deletion of 9p by microarray. Fluorescent in‐situ hybridisation did not identify *MYC* rearrangements typical of Burkitt lymphoma. Targeted molecular studies identified *NRAS* and *BRAF* variants and whole genome and transcriptome analysis demonstrated a *MEF2D::HNRNPUL1* rearrangement (Figure 1, right image). Prior to molecular results being available, the patient was commenced on R‐CODOX‐M/R‐IVAC, achieving complete remission. With full diagnostic information available, consolidation delivered an ALL regimen. This case highlights how, despite a thorough investigation by all appropriate modalities including morphology, immunophenotyping and cytogenetics, the final correct diagnosis may be delayed until the determination of the molecular profile. Further complicating this case, B‐ALL with MEF2D has only recently been described under the category of B‐lymphoblastic leukaemia/lymphoma with other defined genetic abnormalities (WHO, 2022).

**FIGURE 1:**
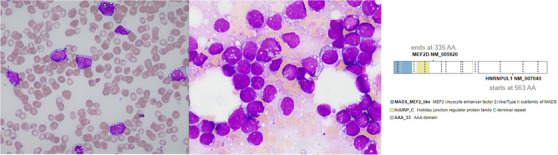
Microscopy of peripheral blood (1000x) (left), bone marrow (1000x) (middle) and schematic of in‐frame MEF2D::HNRNPUL1 fusion transcript (right).

## CONFLICT OF INTEREST STATEMENT

The authors declare no conflict of interest.

## PATIENT CONSENT STATEMENT

Patient has moved overseas so consent is not feasible. Patient's details are adequately anonymised.

## CLINICAL TRIAL REGISTRATION

N/A

## ETHICS STATEMENT

All patient treatment protocols in our institution are agreed to by the Institutional Ethics Committee.

## Data Availability

Data sharing is not applicable to this article as no new data were created or analysed in this study.

